# The match between need and use of health services among healthy under-fives in Denmark: A register-based national cohort study

**DOI:** 10.1371/journal.pone.0231776

**Published:** 2020-04-16

**Authors:** Andreas Jensen, Per Kragh Andersen, John Sahl Andersen, Gorm Greisen, Lone Graff Stensballe

**Affiliations:** 1 Department of Paediatrics and Adolescent Medicine, Rigshospitalet, Copenhagen University Hospital, Copenhagen, Denmark; 2 Section of Biostatistics, Department of Public Health, University of Copenhagen, Copenhagen, Denmark; 3 Section of General Practice and Research Unit for General Practice, Department of Public Health, University of Copenhagen, Copenhagen, Denmark; 4 Department of Neonatology, Rigshospitalet, Copenhagen University Hospital and the University of Copenhagen, Denmark; Federal University of Sergipe, BRAZIL

## Abstract

**Objectives:**

To study a potential positive association (referred to as ‘a match’) between the need for health service (expressed by a mortality risk score) and observed health service utilisation among healthy Danish under-fives. Further, municipal differences in the match were examined to motivate focused comparisons between the organisation of regional health services.

**Design:**

Register-based national cohort study.

**Participants:**

The population of 1,246,599 Danish children born 1997–2016 who survived until date of first discharge to the home after birth without a diagnosis of severe chronic disease.

**Main outcome measures:**

Hazard ratios (HR) for a doubling of the mortality rate were calculated for the following health services: total contacts, inpatient contacts (admission > 1 day), outpatient contacts, general practitioner contacts, specialist contacts, medication use, and vaccinations.

**Results:**

The use of total contacts, inpatient contacts (> 1 day) and general practitioner contacts as well as medication matched with the mortality risk score, HRs between 1.027 (1.026 to 1.028) and 1.111 (1.108 to 1.113), whereas outpatient and specialist contacts as well as vaccinations did not, HRs between 0.913 (0.912 to 0.915) and 0.991 (0.991 to 0.991). There were some remarkable differences among the 98 Danish municipalities.

**Conclusions:**

We found some match between need and use for total contacts, inpatient contacts (> 1 day), contacts with general practitioner, and medication use although the associations were relatively weak. For outpatient and specialist contacts, the mismatch may be related to services not addressing potentially fatal disease whereas for vaccination there was a small mismatch. Our results indicate local discrepancies in diagnosis, and a low adjusted utilisation of hospital admissions in Aarhus compared to the other three major cities in Denmark suggests that a comparison of the organisation of services could be useful.

## Introduction

### Background

During the last quarter of a century the global under-five mortality was more than halved [1]. However, socioeconomic inequalities in child mortality persist between *and* within countries [2,3]. Denmark is a high-income country, but even in the Danish setting inequality may be present despite free access to health services, since more resourceful citizens may be better at seeking health services, or the health care system may not prioritise according to the need of the citizens [4–6].

Attempts to systematise the assessment of the need of care on not only the individual level but also in a population have been made in the past [7], but to our knowledge the literature on the match between need and use of paediatric health services is sparse–especially in high-income settings. However, the research area is not completely uncovered, and one systematic review pointed at *paediatric overutilization* in various situations [8]. These studies analysed decisions regarding treatments against specific diseases (e.g. thresholds) and not overall health service utilisation outcomes in a population.

In a previous Danish national register-based cohort study we examined the temporal development of health service utilisation among Danish under-fives. We observed increased specialisation manifested by increasing use of hospital contacts and specialists and decreasing use of general practitioners among the group of healthy children without severe chronic disease. We interpreted these findings as a potential sign of overdiagnosis [9]. The use of health services among children with chronic disease is high [9]. In the present study we found it particularly relevant to focus on the group of healthy children without chronic disease in order to observe patterns of potential overdiagnosis/overuse.

In another recent study we investigated risk factor profiles for post-discharge under-five mortality with a focus on the group of children not suffering from severe chronic disease [10]. In that study, we found that children with severe chronic diseases had a vastly increased hazard of death—comprising around two thirds of the mortality—which was not surprising given previous results from another high-income setting [11]. However, we also found that certain socioeconomic markers were still associated with mortality among healthy Danish children.

### Objectives

The objective of the present study was to examine the match between need and use by estimating the associations between the child-specific mortality risk factor profiles in healthy Danish under-fives and observed health service utilisation. Data were based on the Danish national health registers [12]. One could argue that survival is the ultimate—albeit not the only—parameter in the assessment of health service. Thus, one would expect a positive association between the estimated mortality risk score and the utilisation of different health services. The opposite—a mismatch—could indicate an element of inexpedient use of resources (overuse), overtreatment or overdiagnosis [13].

Regional or municipal patterns perhaps influenced by local working procedures and recommendations may point out important local differences in the need and use as potential signs of under- or overdiagnosis. In an exploratory analysis, we additionally examined municipal differences in health service utilisation after adjustment for the mortality risk factor profiles of the children in each of the 98 Danish municipalities.

## Methods

### Study design and participants

The present paper describes a register-based national cohort study in Danish children who were followed until five years of age. The background population behind the present study was the cohort of all children live-born in Denmark in the period 1997 to 2016.

The formal exclusion criteria were:

- Death before first discharge from the hospital to the home after birth
- A registered migration before first discharge from the hospital to the home after birth
- A diagnosis of severe chronic disease before first discharge from the hospital to the home after birth [10]
- Not yet discharged from the hospital to the home after birth at 31 December 2016
- Not yet discharged from the hospital to the home after birth at five years of age

The background population of the cohort used in the study was further described in previous studies [9,14].

### Data quality

Data was obtained from the Civil Personal Register [15], which covers the entire Danish population, and from the following national health registers: the Cause of Death Register [16], the Medical Birth Registry [17], the National Patient Register [18], the Register of Medicinal Product Statistics [19], and the National Health Service Register [20]. In addition, registers with information on socioeconomic variables were used: The Population’s Education Register, The Employment Classification Module, The Integrated Database for Labour Market Research and The Income Statistics Register [12]. The variables defined in the section below were all derived from these national registers. It has been concluded that the quality of the data in the Danish registers is high and that the data serve as a valuable tool in epidemiology [15].

### Variables

#### Mortality risk score

Risk factors for post-discharge under-five mortality were analysed in our prior study [21]. The previous study analysed this outcome in the population of healthy children i.e. children not diagnosed with chronic disease based on a previously published register-based algorithm [10]. Time to death was analysed using Cox regression with the pre-selected risk factors included as explanatory variables (cf. Table 1). For each risk factor a separate category was reserved for missing values. The cumulative incidence of childhood mortality was very low cf. the (Graph A in S1 File). After five years the mortality risk was only around seven per 10,000 children, and thus a doubling of the hazard corresponded to an approximate doubling of the absolute risk. Using the estimated coefficients from this model, a ‘mortality risk score’ may be calculated for each child with a given set of risk factors. This is the so-called ‘linear predictor’ in the model and higher values of this score indicate a higher risk of death. In the present study, the estimated linear predictor was thus applied to quantify the mortality risk of each child. The mortality risk score was re-scaled such that one unit increase in the score was associated with a doubling of the hazard of death. The estimated coefficients behind the mortality risk score are presented in the (Table A in S1 File). The concordance between predicted and observed survival times of the mortality risk model was evaluated using Harrell’s C. The result of C = 0.87 indicated a strong model fit [22]. The score was used as surrogate measure of healthcare need in the present study.

**Table 1:** Distribution of predictors used in the calculation of the child-specific mortality risk score.

Predictor	Levels (%)	Missing percentage (*n*)
Maternal age	< 25 years (12.94), 25–29 yrs (33.32), 30–35 yrs (40.18), > 35 yrs (13.57)	0.00% (54)
Paternal age	< 25 years (6.01), 25–29 yrs (23.70), 30–35 yrs (41.73), > 35 yrs (27.35)	1.21% (15,046)
Siblings	No (43.44), yes (53.80)	2.76% (34,367)
Ethnicity	Danish (89.52), other (10.22)	0.26% (3,245)
Live with both parents	No (10.57), yes (89.17)	0.26% (3,245)
Maternal education	Primary (0.64), secondary (55.91), tertiary (38.22)	5.22% (65,107)
Paternal education	Primary (0.41), secondary (62.33), tertiary (30.43)	6.83% (85,096)
Maternal job situation	Work (70.82), unemployed (3.36), out of workforce (15.17), student (9.58)	1.06% (13,230)
Paternal job situation	Work (82.32), unemployed (2.43), out of workforce (7.62), student (4.86)	2.77% (34,566)
Family income (*)	Lowest tercile, mid tercile, highest tercile	4.61% (57,432)
Gender	Female (48.78), male (51.22)	0% (0)
Maternal atopic disease	No (95.81), yes (4.19)	0% (0)
Paternal atopic disease	No (96.42), yes (3.58)	0% (0)
Smoking	No (75.62), yes (16.24)	8.14% (101,417)
Gestational age (GA)	< 28 weeks (0.08), 28–37 weeks (6.62), > 37 weeks (91.15)	2.15% (26,826)
Birth weight	< 2500 g (4.66), ≥ 2500 g (93.52)	1.82% (22,719)
Small for GA	No (94.61), yes (2.87)	2.52% (31,413)
Caesarean section	No (80.45), yes (19.55)	0.00% (54)
Multiple birth	No (96.27), yes (3.72)	0.00% (54)
Birth year	*Continuous*: years since 1997	0% (0)

(*) The family income variable is relative to the distribution within calendar year.

#### Health service utilisation outcomes

In another of our prior studies we examined health service utilisation among Danish under-fives [9]. The services considered were health care contacts (inpatient, outpatient, general practitioner, specialist, and their total) and medication use. The details regarding these outcomes are described in the reference [9].

The present study was based on the same outcomes. However, due to local differences in the definition of inpatient hospitalisations and the fact that some paediatric departments registered admissions with less than one day of duration as acute *outpatient* contacts, only inpatient contacts with a duration of more than one day were included and denoted as inpatient contacts (> 1 day) [23].

Also, we considered vaccination as an additional health service outcome. Vaccinations differ from the other outcomes, since almost all vaccinations are administered according to the Danish child vaccination programme [24]. In a previous study we analysed markers of socioeconomic status and other variables as determinants for vaccination uptake among Danish children [14], and some of these factors were found to be associated with vaccination status. Thus, we found it relevant to include vaccination as a health service utilisation outcome to examine if the individuals in most need (i.e. high mortality risk score) were vaccinated more frequently. The types of vaccinations considered in the present study are presented in the (Table B in S1 File). All seven main outcomes considered in the present study are presented in Table 2.

**Table 2:** Health service utilisation outcomes each regressed on the mortality risk score.

Outcome	Notes
Total contacts	Inpatient, outpatient, emergency, general practitioner and specialist contacts
Inpatient contacts (> 1 day)	All-cause admissions to hospital with > 1 day between admission and discharge
Outpatient contacts	All-cause outpatient contacts
General practitioner contacts	Visits at the general practitioner including the out-of-hours service
Specialist contacts	Dermatology, ear-nose-throat, ophthalmology, psychiatry/psychology, paediatrics
Prescribed medication	All types of dispensed prescriptions
Vaccinations	Childhood vaccinations administered in general practice [14]

Throughout the paper, the term ‘match’ refers to a positive association between mortality risk score and health service utilisation, whereas a ‘mismatch’ refers to a negative association (or no association).

### Study size

The number of children born alive in Denmark during the study period 1997–2016 was 1,268,222. Before discharge from hospital to the home 3,976 children died, 361 were not yet discharged during the study period, and 90 were registered with a migration before discharge. Further 17,196 children were diagnosed with chronic disease [10]. Thus, 1,246,599 children entered the analysis.

### Statistical methods

The purpose of the study was to investigate the relationship between the hazard of mortality and health service utilisation. The mortality risk score was used as the sole covariate in seven different Cox regression models analysing the following recurrent event outcomes: total contacts, inpatient contacts (> 1 day), outpatient contacts, general practitioner contacts, specialist contacts, dispensed prescribed medication, and vaccinations (cf. Table 2).

Using age as the underlying time scale, all children entering the analysis were followed from date of first discharge from the hospital to the home and until death, migration, diagnosis with severe chronic disease, 31 December 2016 or five years of age. In the present study the children with chronic disease were censored at the date of diagnosis to assess potential overuse among otherwise healthy children.

Children remained in the risk set after each occurrence of the event of interest. The dependence between recurrent events for the same child was accommodated by applying a clustered sandwich estimator.

#### Municipal differences

For each of the seven recurrent event models described in Table 2 the martingale residual was calculated for each child. Then for each of the 98 Danish municipalities these martingale residuals were averaged to obtain municipality-specific residuals. In each municipality, these residuals were thus mean deviations from the expected number of events given the mortality risk score.

The following statistical packages were used to analyse the data: R version 3.5.2, SAS 9.4, and Stata/MP 15.1.

### Ethics statement

Data were secured within the servers of Statistics Denmark and analysed via a safe password-protected online data access. All data were fully pseudonymised. According to Danish law, neither ethics committee approval nor patient consent is required for register-based studies.

## Results

### Mortality risk score

**Fig 1:**
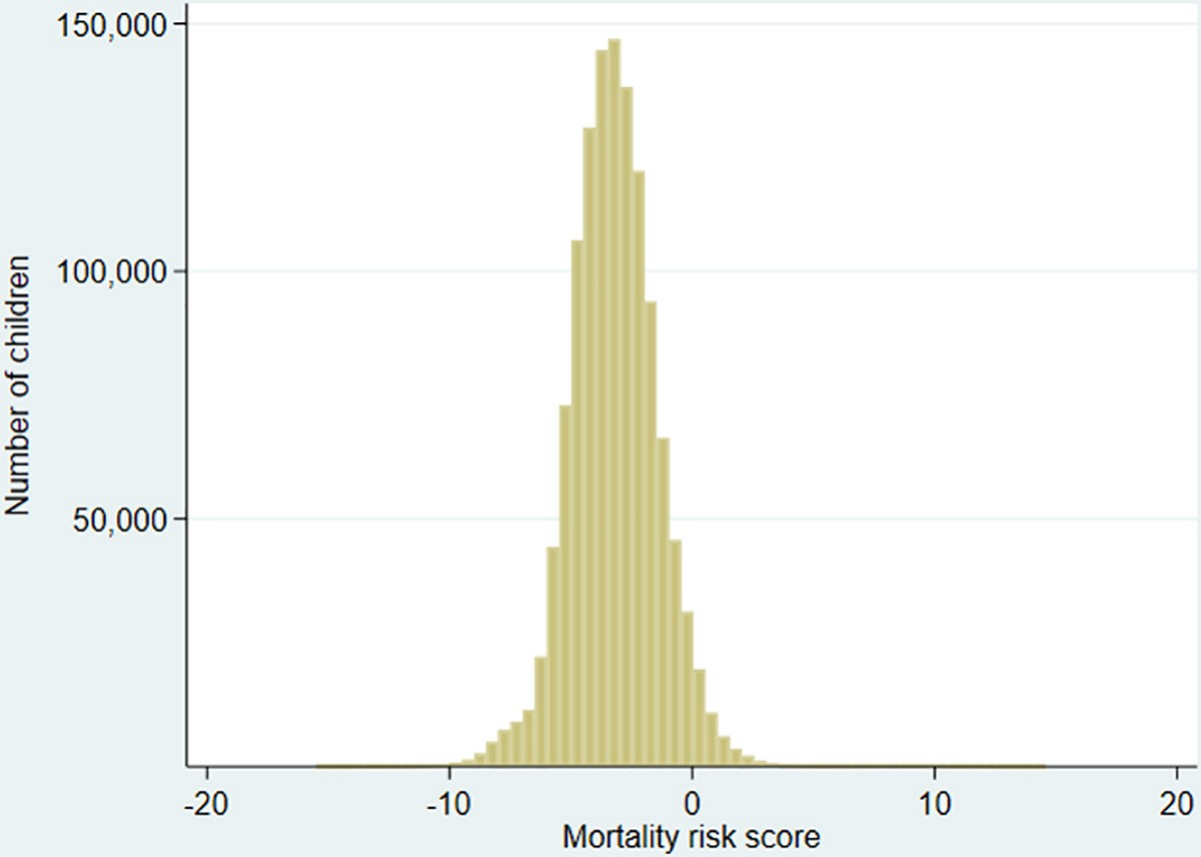
Histogram of the mortality score among the 1,246,599 children entering the analysis.

The distribution of the mortality risk score estimated from the Cox model of the original paper [21] is visualised in Fig 1. Higher scores indicate a higher mortality risk with an increase by 1 unit representing a doubling of the hazard of death.

It is seen from Fig 1 that the mortality scores were distributed in an approximate range from -20 to 20 with 95% of the children having a score between -6.79 and +0.44.

### Match between need and use of health services

In each analysis a child remained in the risk set after each event occurrence and were censored at death (*n* = 719), emigration (*n* = 33,512), diagnosis with severe chronic disease (*n* = 44,894), 31 December 2016 (*n* = 271,234), or five years of age (*n* = 896,240). The total amount of follow-up time for the 1,246,599 children was 5,244,695 person-years i.e. around 4.21 years of follow-up per child on average.

The analyses assumed a linear relationship between the mortality score and the log-hazard of the recurrent events. Two graphical assessments exemplified by the outcome of inpatient contacts, indicated that the linearity assumption was tenable cf. (Graphs B and C in S1 File).

The results of the recurrent event analyses are summarised in Table 3.

**Table 3:** Hazard ratio (robust 95% CI) for the mortality score and seven types of health services until 5 years of age.

Outcome	Hazard ratio (robust 95% CI)	Number of recurrent events	Mean number of events per child until 5 years	Mean number of events per year
Total contacts	1.027 (1.026–1.028)	57,107,916	45.81	9.16
Inpatient contacts (> 1 day)	1.111 (1.108–1.113)	496,124	0.40	0.08
Outpatient contacts	0.913 (0.912–0.915)	1,401,847	1.12	0.22
General practitioner contacts	1.039 (1.039–1.040)	43,822,655	35.15	7.03
Specialist contacts	0.981 (0.979–0.982)	10,270,031	8.24	1.65
Prescribed medication	1.051 (1.050–1.052)	12,865,893	10.32	2.06
Vaccinations	0.991 (0.991–0.991)	7,672,652	6.15	*

(*) Vaccinations are mainly administered at designated ages according to the Danish child vaccination programme.

To enable alternative analyses the mortality risk score was defined in two other ways. First, the score was calculated from a model in which only deaths from natural causes were considered as events (i.e. deaths from external causes (injury, poisoning etc.) were formally censored) [25]. Second, the score was calculated in the population including children with chronic disease where chronicity at first discharge from the hospital to the home after birth was included as a factor in the calculation of the mortality risk score. In both cases the recurrent event outcomes presented above were analysed in the relevant populations using the alternative score. The hazard ratios from these analyses are presented in the (Table C in S1 File): in the first alternative analyses excluding deaths from non-external causes, the hazard ratios did not change noticeably compared to those of the original analysis, but in the second alternative analyses including children with chronic disease in the population, the estimates generally pointed towards a stronger match between need and use (Table C in S1 File).

The chronic diagnoses observed most frequently are presented in the (Table D in S1 File).

According to the model, a doubling of the hazard of death was for example associated with a 2.7% higher rate of total contacts (Table 3). On the other hand, a doubling of the hazard of death was associated with an 8.7% lower rate of outpatient contacts.

When analysing all inpatient contacts (also those < 1 day, n = 885,554) against the mortality risk score, the hazard ratio was only 1.050 (robust 95% CI: 1.048 to 1.052) compared to 1.111 (1.108 to 1.113) for the longer inpatient contacts, which was not surprising given that shorter contacts were generally thought to be less serious. The variation between municipalities was slightly larger when considering all inpatient contacts and the map is presented in the (Fig A in S1 File).

### Municipal differences

**Fig 2:**
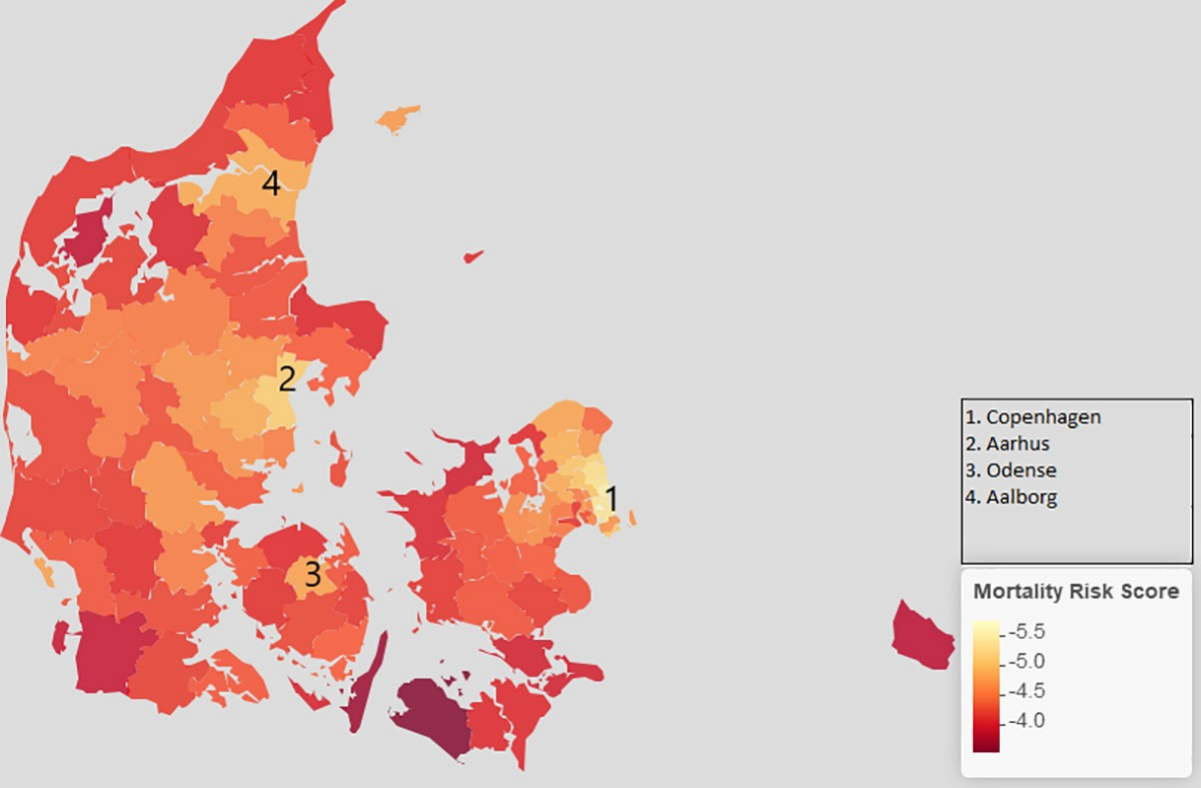
Mean mortality risk score across the 98 municipalities of Denmark–darker colours indicate higher mortality scores. The municipalities containing the four largest cities are marked with numbers 1–4.

For the exploratory analysis of municipal differences in match between need and use of health services 3,245 children (0.26%) were excluded due to missing information on municipality. Fig 2 visualises the mean mortality risk scores across the 98 Danish municipalities with darker colours representing a composition of children with a higher average mortality risk score (and vice versa). This serves as background information on the risk profiles in each municipality. The analyses were not (directly) dependent on geography, and thus the averaged martingale residuals were not necessarily zero within municipalities (as they were in the entire child population per definition). From Fig 2 it is seen that even in the child population of the Danish high-income society, clear regional differences in risk profiles exist. The municipalities containing the four largest cities Copenhagen (the capital), Aarhus, Odense and Aalborg were characterised by child populations with low mortality risk scores.

**Fig 3:**
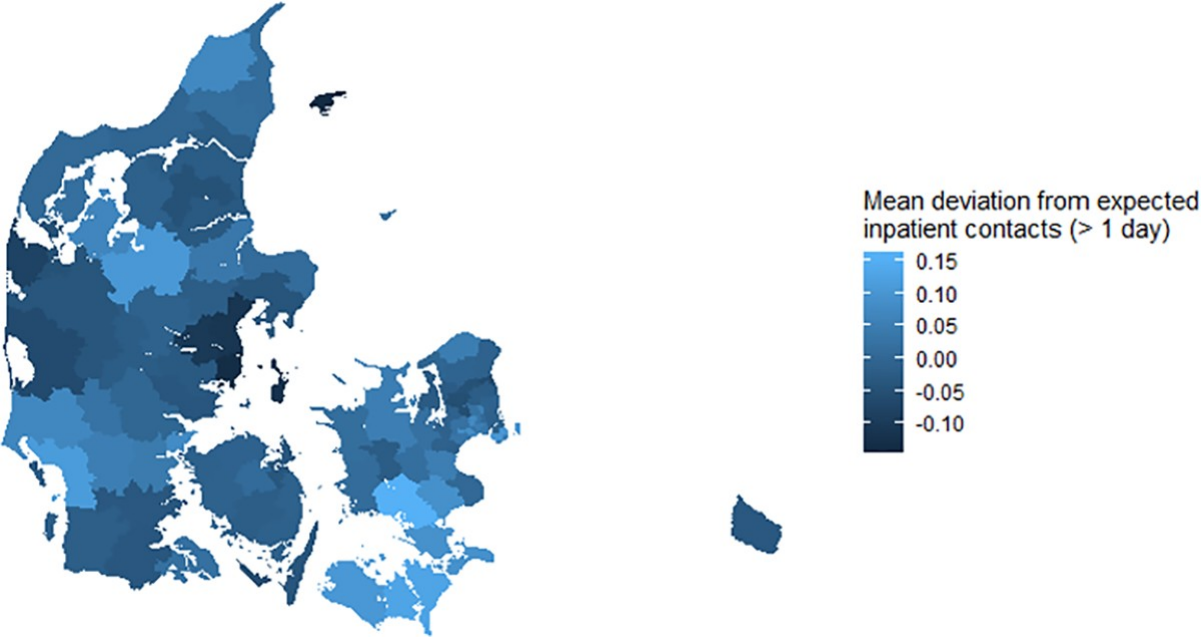
Mean deviation from the expected number of inpatient contacts (> 1 day) in Danish children until 5 years of age across the 98 municipalities–darker colours indicate lower consumption.

Fig 3 shows the mean residuals for inpatient contacts (> 1 day) within municipalities. These residuals are deviations from the expected number of inpatient contacts (> 1 day) under the recurrent events model regressed on the mortality risk score. Similar figures displaying municipal differences for the remaining outcomes (including inpatient contacts of all durations) can be found in the (Figs A-G in S1 File).

Fig 3 shows whether the children in the respective municipalities on average experience more or fewer inpatient contacts (> 1 day) than expected considering their mortality risk score profile.

The urban area of Aarhus appeared to differ markedly from the rest of the country (cf. the Discussion section). The children in Aarhus had on average a 28.09 percent lower rate of inpatient contacts (> 1 day) than expected, whereas the corresponding mean deviation of the three other large municipalities was only 1.91 percent below the expected rate.

Table 4 lists–for all seven outcomes—the range of deviations from the expected number of events across the 98 municipalities, and, in addition, these absolute deviations are translated to percentages from the mean number of the given event type among all the children until 5 years of age (see Table 3).

**Table 4:** Municipal range of deviations from expected health service utilisation until 5 years of age in the 98 municipalities.

Outcome	A) Range of deviations from expected absolute number of events	B) Range of deviations from national mean number of events (%)
Total contacts	-18.87 to +8.47	-41.20% to +18.50%
Inpatient contacts (> 1 day)	-0.14 to +0.15	-35.02% to +38.69%
Outpatient contacts	-0.95 to +0.43	-84.34% to +38.24%
General practitioner contacts	-9.77 to +6.85	-27.79% to +19.49%
Specialist contacts	-7.61 to +4.10	-92.42% to +49.71%
Prescribed medication	-5.78 to +3.26	-56.02% to +31.60%
Vaccinations	-0.48 to +0.29	-7.80% to +4.79%

A) Deviations averaged among all children in the municipality; B) the deviations in A) relative to the national mean.

The wide ranges presented in Table 4 indicate large discrepancies within and between the outcomes. The relatively small range for vaccination is explained by the fact that this outcome primarily follows the Danish child vaccination programme.

## Discussion

The present study analysed the match between the need and use of health services among Danish under-fives without severe chronic disease from 1997 to 2016.

Overall, we found a match for total contacts: 1 unit increase in mortality score (doubled hazard of death) was associated with a 2.7% higher rate of experiencing a contact. Thus, health services seemed sensitive to the children in most need e.g. premature children or children of families with low socioeconomic status, but the ability to compensate for higher mortality risk in the form of increased health service was limited. However, note that neither the mortality score as a measure of need nor the health service utilisation as a measure of provision are perfect. Indeed, it is not obvious what an appropriate match would be.

A match was found for inpatient and general practitioner contacts and medication use for which the associations with the mortality risk score were increased rates of 11.1%, 3.9%, and 5.1%. Conversely, for outpatient and medical specialist contacts and vaccinations, the mortality risk score was associated with decreased rates. For outpatient contacts 1 unit increase in mortality risk score was associated with an 8.7% lower rate of contacts. This may suggest that healthy children who are able to wait for service use outpatient and medical specialist services which may not be an optimal spend of resources. In another study we found increasing rates of outpatient and medical specialist contacts in healthy children [9] which are potential signs of overdiagnosis/overuse [8]. On the other hand, specialist services may be directed at non-lethal problems, e.g. vision, hearing, or the skin, occurring often in under-fives. For vaccinations the association with mortality risk score was not surprising since–even in children without chronic diagnoses–healthier children with higher socioeconomic status are more likely to be vaccinated [14,26].

A clear rural-urban gradient was found. The mortality risk score was low in the four large urban areas in Denmark reflecting high socioeconomic status of urban families. However, marked differences between the four major urban areas were visible. In Aarhus children on average had fewer inpatient contacts than expected under the model. This contrasted with higher use of inpatient contacts in the other large cities with similar risk profiles. The time period of the present analysis included decreasing mortality in healthy Danish children, and steady total use but increased specialisation of health services [5,8,9]. Thus, a focused comparison of the organisation of the health system responsible for referral and admission of ill, but basically healthy children in the four major urban areas in Denmark could be useful: perhaps Copenhagen admit too many children or, alternatively, Aarhus admit too few.

Mortality is not the only endpoint of interest and the health system should not only be designed to prevent deaths, but also to target conditions not ultimately resulting in death. In addition, the demand for health service is patient-driven, and the health care system is not solely responsible for the consumption. Finally, the children in most need have chronic diseases. This study primarily concerned healthy children, since chronic children would most likely inflate both mortality risk score and health service utilisation [9] thus imposing an association, but local differences could be explored in this population too. As expected, the alternative analysis which included children with chronic disease indeed revealed a stronger match between the need and use of health services. For example, the hazard ratio for inpatient contacts (> 1 day) was 1.22 when including all children compared to 1.11 in the healthy population. This indicates that the hospital system tends to admit children with high mortality risk and chronic disease even more than it is the case for otherwise healthier children with relatively high mortality risk. This suggest that this part of the hospital system appropriately undertakes a holistic view considering social and medical aspects.

The analysis of over- or under-utilisation was also limited by the absence of a benchmark for appropriate levels of health service utilisation. A positive association between mortality risk score and increased utilisation (e.g. for general practitioner contacts) does not imply that over- or under-utilisation was present. Thus, one could argue that the results of the analyses in which the need was associated with lower utilisation were the most interesting (e.g. outpatient contacts).

While there is clearly room for other methodologies and perspectives, the present study does suggest a relatively simple approach to study the match or mismatch between need and use of health services.

## Conclusion

Using a simple study approach comparing mortality risk scores with health service utilisation among healthy Danish under-fives, we found some match between need and use for the outcomes of total contacts, inpatient contacts (> 1 day), contacts with general practitioner, and medication use, and a mismatch for outpatient contacts, medical specialist contacts and vaccinations. The mismatch for outpatient contacts and medical specialists may indicate that these services are not related to fatal disease. Our results may indicate national and local signs of overuse/overdiagnosis and call for a discussion of organisation and procedures behind paediatric health services in Denmark.

## Supporting information

S1 File (DOCX)
