# Supplementary material for: The match between need and use of health services among healthy under-fives in Denmark: A register-based national cohort study
Source: PLoS One. 2020 Apr 16;15(4):e0231776. doi: 10.1371/journal.pone.0231776 (PMC7161958; doi:10.1371/journal.pone.0231776)
Supplement: S1 File — (DOCX) [file pone.0231776.s001.docx]

**Graph A in S1 File. Cumulative baseline mortality risk.**

**
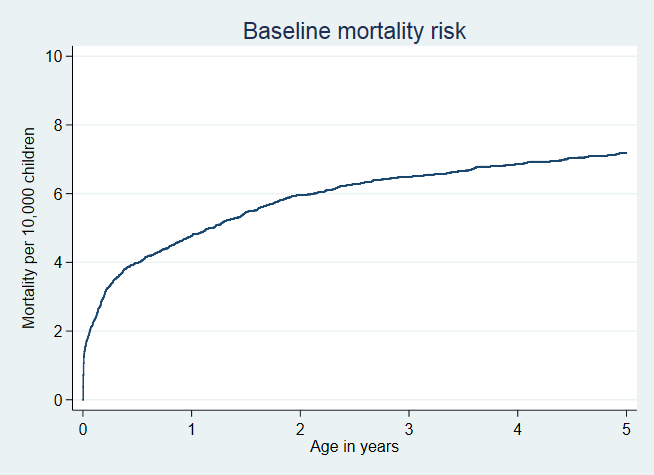
**

**Table A in S1 File. Coefficients in mortality risk score.** Coefficient is zero for the reference level.

| **Risk factor (reference level)** | **Levels** | **Estimated coefficient (95% CI)** |
| --- | --- | --- |
| Maternal age (ref: < 25 years) | 25-29 years | -0.57 (-0.79 to -0.35) |
|  | 30-35 years | -0.77 (-1.02 to -0.51) |
|  | > 35 years | -0.59 (-0.92 to -0.26) |
| Paternal age (ref: < 25 years) | 25-29 years | -0.15 (-0.42 to 0.13) |
|  | 30-35 years | -0.28 (-0.58 to 0.01) |
|  | > 35 years | -0.10 (-0.43 to 0.26) |
| Siblings (ref: no) | Yes | 0.21 (0.03 to 0.39) |
| Ethnicity (ref: Danish) | Non-Danish | 0.26 (-0.08 to 0.60) |
| Living with both parents (ref: no) | Yes | -0.40 (-0.67 to -0.13) |
| Maternal education (ref: primary) | Secondary | -0.17 (-0.90 to 0.55) |
|  | Tertiary | -0.39 (-1.13 to 0.36) |
| Paternal education (ref: primary) | Secondary | 0.25 (-0.65 to 1.15) |
|  | Tertiary | -0.11 (-1.03 to 0.80) |
| Maternal job situation (ref: employed) | Unemployed | 0.37 (0.05 to 0.68) |
|  | Outside workforce | -0.17 (-0.37 to 0.03) |
|  | Student | -0.32 (-0.62 to -0.02) |
| Paternal unemployment (ref: employed) | Unemployed | 0.00 (-0.38 to 0.37) |
|  | Outside workforce | -0.24 (-0.49 to 0.00) |
|  | Student | -0.66 (-1.08 to -0.25) |
| Family income (ref: lowest tertile) | Mid tertile | -0.28 (-0.56 to 0.00) |
|  | Highest tertile | -0.52 (-0.88 to -0.16) |
| Gender (ref: female) | Male | -0.03 (-0.18 to 0.12) |
| Maternal atopic disease (ref: no) | Yes | 0.13 (-0.19 to 0.45) |
| Paternal atopic disease (ref: no) | Yes | -0.16 (-0.58 to 0.27) |
| Smoking (ref: no) | Yes | 0.72 (0.54 to 0.89) |
| Gestational age (GA) (ref: > 37 weeks) | 28-37 weeks | 0.00 (-0.31 to 0.31) |
|  | < 28 weeks | 1.22 (0.65 to 1.79) |
| Birth weight (ref: ≥ 2500 g) | < 2500 g | 0.55 (0.19 to 0.92) |
| Small for GA (ref: no) | Yes | 0.19 (-0.14 to 0.53) |
| Caesarean section (ref: no) | Yes | 0.21 (0.02 to 0.40) |
| Multiple birth (ref: no) | Yes | 0.26 (-0.08 to 0.59) |
| Birth year (ref: 1997) | Per year since 1997 | -0.10 (-0.12 to -0.09) |

**Text A in S1 File. Vaccination codes.**

The vaccination codes from the variable *SPECIALE* in the Danish National Health Service Register [1] used in the definition of the vaccination outcome considered in the main paper are presented in Table S1.

**Table B in S1 File. List of vaccinations from the National Health Service Register considered for the vaccination outcome.**

| **Type of vaccination** | **Codes** |
| --- | --- |
| Diphteria-Tetanus-Pertussis-Polio-Haemophilus Influenzae B | 808341-808343 |
| Diphteria-Tetanus-Polio | 258321-258323, 808321-808323 |
| Diphteria-Tetanus-Pertussis | 808324 |
| Diphteria-Tetanus-Pertussis-Polio | 808325 |
| Pertussis | 258311-258313, 808311-808313 |
| Oral polio | 258331-258333, 808331-808333 |
| Haemophilus Influenzae B | 258501-258503, 808501-808503 |
| Diphteria-Tetanus | 484130 |
| Tetanus | 484275 |
| Measles-mumps-rubella | 258601, 808601-808617, 838612 |
| Measles | 484101 |
| Pneumococcal | 484235, 484283, 808344-808346 |
| Infanrix Hexa | 808350-808354 |
| Various other vaccinations ^(*)^ | 484106, 484108, 484112-484129, 484131-484219, 484226, 484230, 484276-484280, 484282, 484284-484287, 489908, 489914, 804101-804104, 808314-808320, 808326, 808327, 808801 |

(*) Details: <https://www.dst.dk/da/TilSalg/Forskningsservice/Dokumentation/hoejkvalitetsvariable/sygesikring---ydelser/speciale>

**Graph B in S1 File. Martingale residual plot for the inpatient outcome.**

**
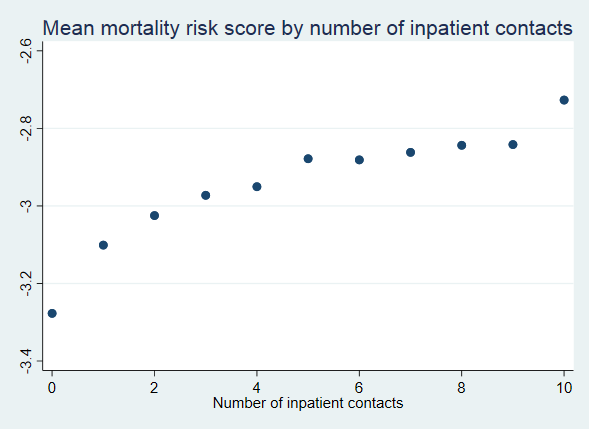
**

Restricted to 10 contacts, since for larger values the mean mortality risk scores were based on few children.

**Graph C in S1 File. Hazard ratios for inpatient contacts analysed using mortality risk score deciles.**


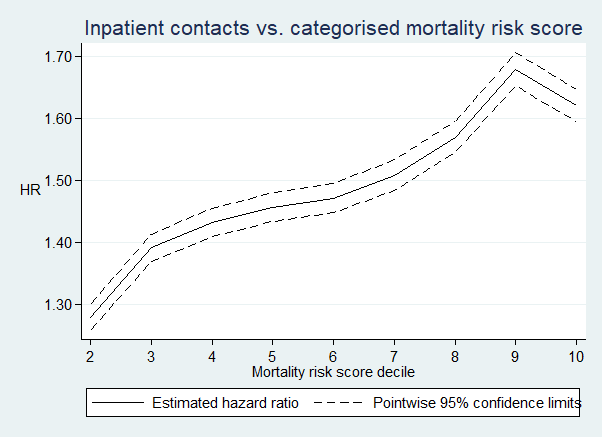


**Table C in S1 File. Hazard ratio (HR) with robust 95% CI for health services using different versions of mortality scores**

| **Outcome** | **HR (95% CI) mortality score based on non-external causes of death** | **HR (95% CI) including chronic disease at first discharge in the score** |
| --- | --- | --- |
| Total contacts | 1.026 (1.025-1.026) | 1.046 (1.045-1.047) |
| Inpatient contacts (> 1 day) | 1.108 (1.105-1.110) | 1.220 (1.216-1.224) |
| Outpatient contacts | 0.914 (0.912-0.916) | 0.987 (0.984-0.991) |
| General practitioner contacts | 1.037 (1.037-1.038) | 1.056 (1.055-1.057) |
| Specialist contacts | 0.982 (0.981-0.984) | 0.990 (0.988-0.992) |
| Prescribed medication | 1.050 (1.049-1.051) | 1.086 (1.084-1.088) |
| Vaccinations | 0.991 (0.991-0.992) | 0.986 (0.986-0.986) |

**Table D in S1 File. Occurrence of chronic diagnoses.**

| *In total 62,640 children were diagnosed with chronic disease* ^(*)^ | **Number of children diagnosed** | |
| --- | --- | --- |
| **Category of chronic diagnoses** | **Congenital** | **Acquired** |
| Malformations of respiratory tract | 2,972 | . |
| Cystic fibrosis | 277 | . |
| Bronchopulmonary dysplasia | 1,820 | . |
| Other conditions associated with respiratory symptoms | 7,317 | 572 |
| Malformations of the nervous system | 2,138 |  |
| Spinal muscular atrophy, muscular dystrophy and congenital disturbances of muscle tonus | 2,340 |  |
| Cerebral palsy | 3,429 | . |
| Other neuromuscular diseases | 736 | 8,218 |
| Heart disease | 16,263 | 3,557 |
| Renal malformations | 5,338 | . |
| Parenchymatous renal disease and others affecting renal function | 91 | 704 |
| Cancer | . | 1,231 |
| Down’s syndrome | 919 | . |
| Other chromosomal abnormalities | 1,718 | . |
| Other malformations | 6,233 | . |
| Others | 4,336 | 5,694 |

(*) The same child could be diagnosed with more than one condition.

**Fig A in S1 File. Mean deviation from the expected number of inpatient contacts (including those < 1 day) in Danish children until 5 years of age across the 98 municipalities – darker colours indicate lower consumption.
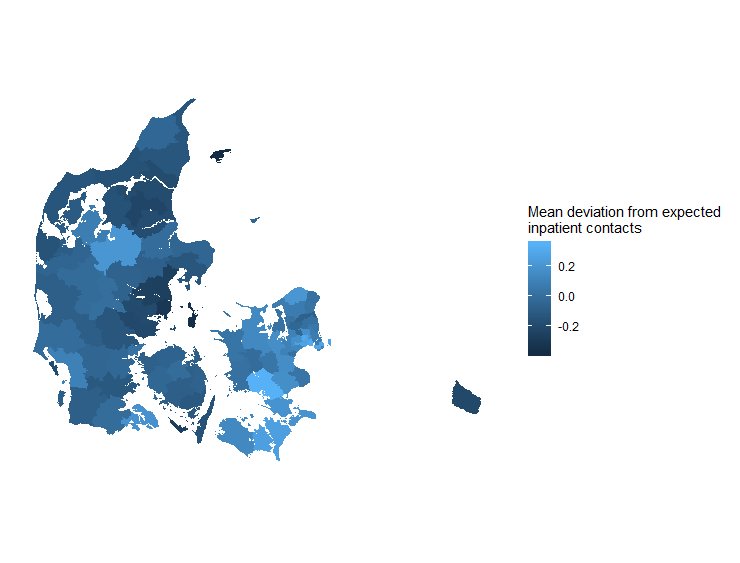
**

**Fig B in S1 File. Mean deviation from the expected number of total contacts in Danish children until 5 years of age across the 98 municipalities – darker colours indicate lower consumption.
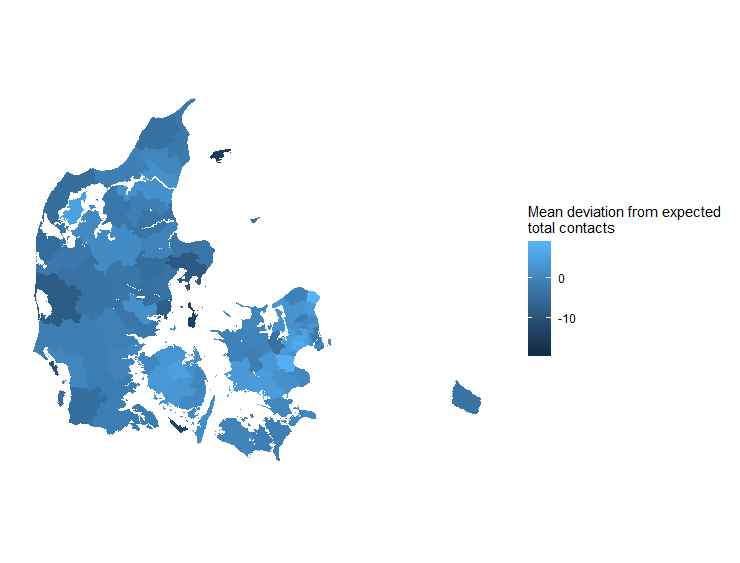
**

**Fig C in S1 File. Mean deviation from the expected number of outpatient contacts in Danish children until 5 years of age across the 98 municipalities – darker colours indicate lower consumption.
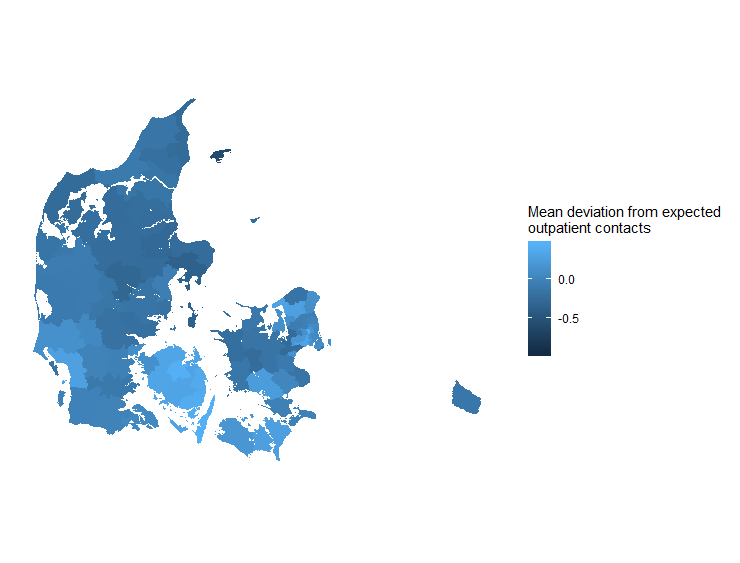
**

**Fig D in S1 File. Mean deviation from the expected number of general practitioner contacts in Danish children until 5 years of age across the 98 municipalities – darker colours indicate lower consumption.
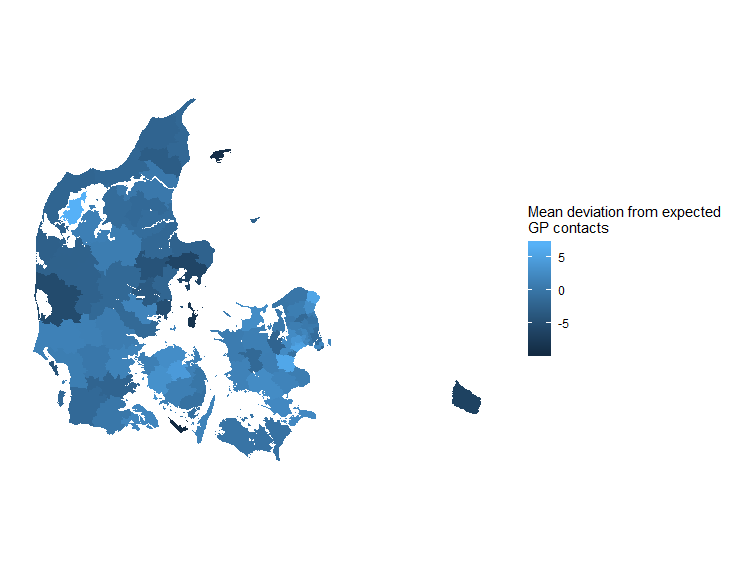
**

**Fig E in S1 File. Mean deviation from the expected number of medical specialist contacts in Danish children until 5 years of age across the 98 municipalities – darker colours indicate lower consumption.
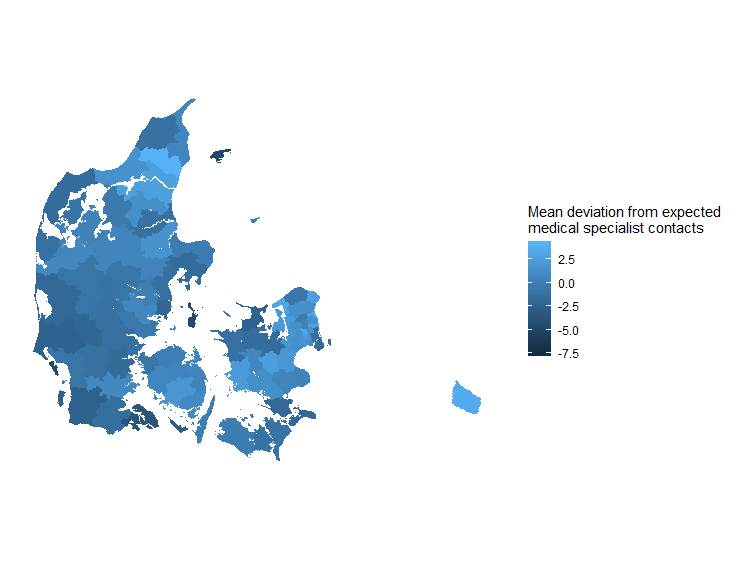
**

**Fig F in S1 File. Mean deviation from the expected number of prescriptions in Danish children until 5 years of age across the 98 municipalities – darker colours indicate lower consumption.
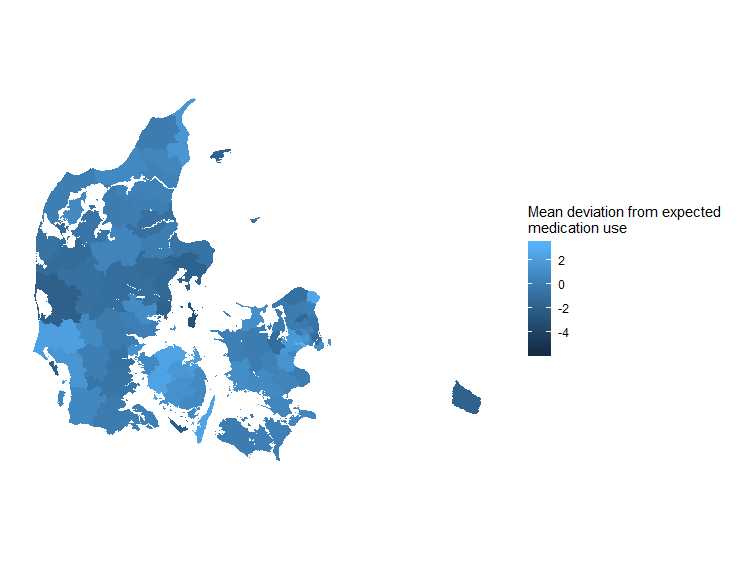
**

**Fig G in S1 File. Mean deviation from the expected number of vaccinations in Danish children until 5 years of age across the 98 municipalities – darker colours indicate lower consumption.
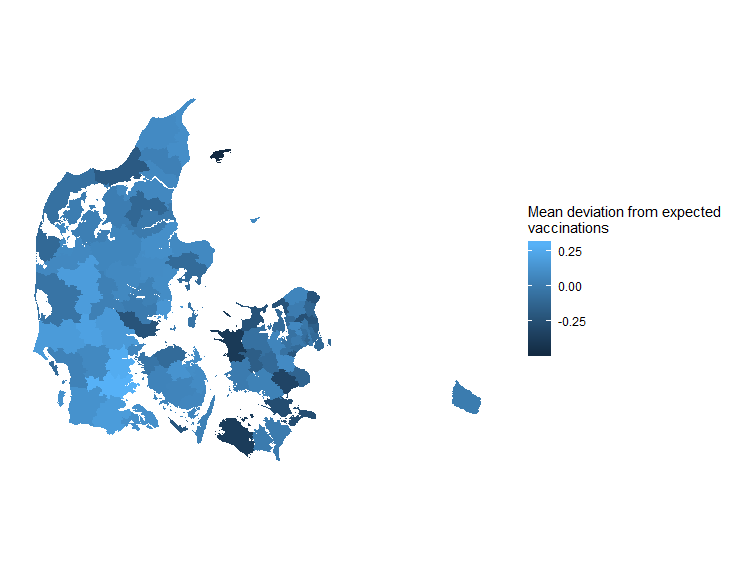
**

**Appendix A in S1 File. Supporting reference.**

1 Andersen JS, Olivarius Ndf, Krasnik A. The Danish National Health Service Register. *Scand J Public Health* [Internet]. 2011;39(7 Suppl):34–7. Available from: http://sjp.sagepub.com/cgi/doi/10.1177/1403494810394718
